# Enhancing the Quality of Black Bean by *Ganoderma oregonense* Solid-State Fermentation and Its Application in Steamed Bread

**DOI:** 10.3390/foods15091588

**Published:** 2026-05-04

**Authors:** Shaojun Zhang, Zhao Wang, Xiaoqin Ren, Kaixu Bai, Hongjun Wang, Yu Wang, Bing Deng

**Affiliations:** 1 College of Food Science and Engineering, Shanxi Agricultural University, Taigu 030801, China; 2 Shanxi Center of Technology Innovation for Storage and Processing of Fruit and Vegetable, Taigu 030801, China; 3 Shanxi Branch, China Grain Reserves Group Ltd., Taiyuan 030000, China

**Keywords:** solid-state fermentation, *Ganoderma oregonense*, ergosterol, fermented black bean flour, steamed bread quality

## Abstract

Rapid population growth has placed increasing pressure on global food systems. As a result, developing healthier and more sustainable food products has become an important research priority. In this context, edible fungi-based fermentation of cereal and legume substrates has attracted growing attention because it can enhance flavor, nutritional value, and bioactive potential through biotransformation. However, the dynamic quality changes of black bean during fungal solid-state fermentation (SSF) and its subsequent application in steamed bread remain insufficiently understood. This study investigated the effect of solid-state fermentation (SSF) using *Ganoderma oregonense* at different durations (12, 19, 26, 33, and 40 d) on the bioenhancement of nutritional and antioxidant activities in black bean. *FTIR* results showed that SSF of *G. oregonense* released active components mainly by destroying its dietary fiber structure. At 40 days of *G. oregonense* fermentation, the total dietary fiber content of black bean declined from 17.09 to 12.98 g/100 g, and the content of reducing sugars increased 5.20 times. *G. oregonense* is outstanding in optimizing protein and free amino acids, and the total amount of protein, γ-aminobutyric acid, essential amino acids, and free amino acids were enhanced by 24.07%, 35.25%, 68%, and 56.74%, respectively. In addition, the fermentation time had a significant effect (*p* < 0.05) on the antioxidant capacity of black bean, with a significant increase in the content of VE, total phenols, and total flavonoids, as well as the production of new antioxidant components. Steamed bread prepared using *G. oregonense* solidly fermented black bean flour (FBBF) shows better antioxidant activity and sensory acceptability than wheat steamed bread. The results suggest that FBBF has potential as a functional ingredient for value-added food applications.

## 1. Introduction

The Food and Agriculture Organisation of the United Nations (FAO) predicts (2021) that the global population will exceed 9.7 billion by 2050. The environmental and food security problems brought about by rapid population growth make it imperative to transform the global food system into a healthy and sustainable one [1]. Since ancient times, edible fungi have been recognized as a valuable source of natural health products and biologically active ingredients for medicinal and dietary use [2]. Edible fungal mycelium can absorb nutrients from the cereal substrate for its reproduction and, at the same time, biotransform nutrients for flavor improvement while enhancing nutritional and functional properties [3,4]. For example, Yang et al. (2020) [4] investigated the solid-state fermentation of soybean residue (okara) using *Ganoderma lucidum* and found that the fermented product exhibited significant anti-osteoporosis effects. Zhang et al. (2024) conducted a study on the liquid fermentation of soybean protein using edible mushroom mycelia, including *Morchella crassipes*, (*Hedgehog fungus*), and shiitake mushroom, and demonstrated that all three strains improved the flavor and processing characteristics of soybean proteins, with the potential to produce nutritious condiments and flavorful plant-based products [5]. Using edible fungi to ferment grains can provide an important solution for several persistent global issues posed by rapid population growth, including human and livestock malnutrition, hunger, and food security [6].

Black beans are rich in protein; various vitamins and minerals, such as phosphorus, zinc, and magnesium; and other essential nutrients. They also contain many bioactive substances that can promote health status, prevent diseases, and provide adequate energy to consumers by reducing the risk of developing some cancers, heart disease, diabetes, and other serious health problems [7]. Due to their beneficial medicinal, food, and therapeutic properties, they are widely used in the food and medical industries to develop functional foods and recover bioactive substances that have physiological effects on living organisms [8]. Several studies have shown that the compounds present in black bean are suitable for biotransformation. Luo et al. (2020) [9] investigated the growth characteristics of edible fungal mycelia on different cereal substrates using seven types of cereals as culture media. Their results showed that the black bean-based medium was the most suitable for *Ganoderma lucidum* mycelial growth, exhibiting dense and uniform mycelia, the highest growth score, and the highest mycelial growth index [9]. Lee I. H. et al. (2008) found that black bean fermentation using *Aspergillus awamori*, *Aspergillus oryzae*, *Aspergillus sojae*, *Rhizopus azygosporus*, and *Rhizopus sp*. significantly enhanced the antioxidant activity of all fermented samples [10]. Furthermore, Lee S. et al. (2018) reported that germinated black bean products fermented with *Lactobacillus pentosus* SC65 possessed significant immunostimulatory activity [11]. Prior to the start of the experiment, the nutritional components of the black beans were analyzed; please refer to Table S1 in the appendix for details.

*Ganoderma lucidum* is one of the most researched and product-developed edible fungi. Refs. [12,13] reported enhanced nutritional and antioxidant activity of black beans solidly fermented with *Ganoderma spp*. and *Coriolus versicolor*. However, studies on the changes in black bean quality during *G. lucidum* fermentation are still quite limited and remain to be fully documented. In addition, the feasibility of *G. lucidum* fermented black bean for functional food development has not been reported. Therefore, in this study, SSF with *Ganoderma oregonense* was used to treat black bean, and the changes in nutrient composition, bioactive compounds, and antioxidant activity of samples at different fermentation times were tracked. In addition, fermented black bean flour (FBBF) was mixed with wheat flour to prepare steamed bread (SB), and the different additions effects of *G. oregonense*-fermented black bean flour on the quality of steamed bread were compared in terms of color, texture, and sensory aspects. The main objective is to clarify the changes and intrinsic correlation of each nutrient component in the process of SSF of black bean with *G. oregonense*, as well as to explore the feasibility of its use in the production of functional foods and the people’s acceptance of this novel food product, in the expectation of providing theoretical guidance for the research and development of related products using SSF of black bean with *G. oregonense* as a high-value raw material.

## 2. Materials and Methods

### 2.1. Materials

Black bean was obtained from a local supermarket (Taigu, Shanxi, China) and stored at 4 °C. *Ganoderma oregonense* was acquired from Jiangsu Tianda Mushroom Research Institute (Yangzhou, Jiangsu, China) and used as a starter for the processing of fermented black bean.

### 2.2. Preparation of Fermented Black Bean (FBB)

Cleaned black bean was mixed with distilled water in proportion (1:4 *w*/*v*) and soaked for 24 h at 25 ± 2 °C. After soaking for 24 h, the moisture content of black beans reached approximately 55%. The fermentation of *G. oregonense* was conducted according to Zhang et al. (2024). A total of 40 glass bottles (7 × 10 × 5.8 cm), each containing 100 g of black bean substrate, were prepared for solid-state fermentation without forced ventilation [14]. The substrate was autoclaved at 121 °C for 120 min and cooled to room temperature prior to inoculation. Three to four pieces of activated *G. oregonense* (0.5 × 0.5 cm) were inoculated into the center of each bottle and incubated at 28 °C. A total of eight sampling groups were included, consisting of one initial control group (0 h) and seven fermentation time points (12, 19, 26, 33, 40, 47, and 54 d). At each sampling point, five bottles were randomly selected as biological replicates for destructive sampling, and the entire contents (including mycelium and substrate) were collected for analysis. The fermented black bean samples were dried to constant weight in a blast drying oven at 40 °C. The dried samples were milled (SM-3C, Hsiang Tai, China) and sieved (screen diameter 200 μm) to produce fermented black bean flour. The flour was stored at −80 °C until further analysis. Non-fermented black bean (NFBB) was subjected to the same treatment as a control.

### 2.3. Determination of Ergosterol Content

Briefly, 0.3 g (dry weight) of flour was soaked in 2 mL ethanol overnight. After centrifugation at 6000× *g* for 20 min, the supernatant was collected. The residue was re-extracted with 2 mL of ethanol using ultrasonic treatment for 40 min, and the supernatants were combined. The ergosterol content was determined by HPLC using a Zorbax C18 column (4.6 × 250 mm, 5 μm, Agilent, Beijing, China) at 35 °C, with 100% methanol as the mobile phase. Detection was performed at 282 nm using a diode-array detector (DAD, Agilent) maintained at ambient temperature [15].

### 2.4. Basic Chemical Composition

The following methods were used to determine reducing sugars, protein, fat, ash, vitamin E and dietary fiber. Reducing sugar content was determined by the 3,5-dinitro salicylic acid method [16]. Crude protein, crude fat and ash content were determined using AOAC 920.87, AOAC 920.85 and AOAC 923.03, respectively. Vitamin E content was determined using a vitamin E content test kit (Solarbio, Beijing, China). The extraction of dietary fiber was carried out using the method of Goñi et al. (2009) [17] and determined according to the gravimetric method described by the national standards of China (GB5009.88-2023: Determination of dietary fiber in food) [18].

### 2.5. Determination of β-Glucosidase Activity

β-Glucosidase activity was determined according to the method of Y. Chen et al. (2020) [19]. Briefly, 0.1 mL of enzyme solution was mixed with 0.8 mL of 100 mM acetate buffer (pH 5.0, Shanghai, China) and 0.1 mL of 5 mM p-nitrophenyl-β-D-glucoside (pNPβG, Shanghai, China). After 30 min incubation at 50 °C, 2 mL of 1 M Na_2_CO_3_ solution was added to stop the reaction, and the released p-nitrophenol was measured at 405 nm. One unit (U) of β-glucosidase activity was defined as the amount of enzyme that catalyzed the release of 1 μmol of p-nitrophenol per minute under the assay conditions.

### 2.6. Determination of Total Phenols, Total Flavonoids, and Total Triterpenes

The total phenolic content (TPC) was determined according to the Folin–Ciocalteu method described in the literature [20]. Briefly, 0.2 mL of sample extract was mixed with 2.3 mL of deionized water and 0.5 mL of Folin–Ciocalteu (Tianjin, China) reagent. After 1 min, 2 mL of 75 g/L sodium carbonate solution was added, and the mixture was incubated in the dark at room temperature for 2 h. The absorbance was measured at 760 nm. Results were expressed as mg gallic acid equivalent (GAE) per 100 g dry weight. Total flavonoid concentration (TFC) was measured using the aluminum chloride colorimetric method [21]. Briefly, 1 mL of sample extract was mixed with 0.3 mL of 5% NaNO_2_; after 5 min, 0.3 mL of 10% AlCl_3_ was added, and after another 5 min, 2 mL of 1 M NaOH was added. The absorbance was measured at 510 nm. Results were expressed as mg rutin equivalent (RE) per 100 g dry weight. Total triterpene content (TTC) was determined by the vanillin-acetic acid and perchloric acid colorimetric method with oleanolic acid as the standard [22]. Briefly, 0.5 mL of sample extract was evaporated to dryness, then mixed with 0.3 mL of 5% vanillin-acetic acid solution and 1.0 mL of perchloric acid. The mixture was incubated at 60 °C for 15 min, cooled, and diluted with 5 mL of glacial acetic acid. The absorbance was measured at 548 nm. Results were expressed as mg oleanolic acid equivalent per 100 g dry weight.

### 2.7. Free Amino Acid Analysis

Free amino acids were extracted by suspending 0.2 g of sample powder in 20 mL of 75% ethanol solution. The suspension was stirred thoroughly using a magnetic stirrer for 30 min at 60 °C and then centrifuged (10,000× *g*, 4 °C, 5 min). The supernatant was evaporated to dryness on a rotary evaporator and reconstituted with 2 mL of 0.2 M sodium citrate buffer (pH 5.0). The extract was filtrated through a 0.22 μm membrane filter and collected into an autosampler vial. The sample was subsequently separated and quantified using an automatic amino acid analyzer (Biochrom 30+, Biochrom, UK) [23]. γ-Aminobutyric acid (GABA) was determined by the Berthelot colorimetric method [24].

### 2.8. Antioxidation Assay

The antioxidant activity of the samples was assessed using 2,2-diphenyl-1-picrylhydrazyl (DPPH) assay and electrochemical methods. The DPPH-radical scavenging was studied by following the method reported by R. Zhang et al. (2024) [14]. Briefly, 2 mL of 0.4 mM DPPH solution (dissolved in methanol) was mixed with 2 mL of sample extract. The mixture was shaken vigorously and incubated in the dark at room temperature for 30 min. The absorbance was measured at 517 nm. Electrochemical experiments were performed as described in our previous paper [23]. A glassy carbon electrode (GCE, radius 1.5 mm) was used as the working electrode. A platinum wire was used as a counter electrode, and a silver/silver chloride electrode (saturated potassium chloride) was used as the reference electrode. The sample solution was prepared with 0.1 M sodium phosphate buffer (pH 4.0), and then electrochemical experiments were performed using an electrochemical station (Autolab PGSTAT 302 N, Utrecht, The Netherlands). Cyclic voltammograms were recorded in the potential range from 0 to 1.4 V at a scan rate of 50 mV/s.

### 2.9. Fourier Transform Infrared (FTIR) Spectroscopy Analysis

The change in the molecular structure of samples was determined by the method of reference Zou et al. (2024) [25]. The sample was mixed with dried KBr powder at a ratio of 1:100 (*w*/*w*), and then the powder was pressed into a transparent sheet and analyzed by an FTIR spectrometer (TENSOR 27, Beijing Bruker Science and Technology Co., Beijing, China) with a resolution of 4 cm^−1^ in the wavenumber range of 400–4000 cm^−1^.

### 2.10. Preparation of SB with FBBF Added (FBBF-SB)

Based on dynamic monitoring of the fermentation process, by the 40th day of fermentation, most of the nutritional components in the *G. oregonense*-fermented black bean flour had reached relatively high levels, whilst the content of certain functional amino acids was at its peak; consequently, this sample was selected for use as a functional ingredient in the preparation of steamed bread. *G. oregonense* black bean powder fermented for 40 d was selected for SB preparation. The recipe for the dough consists of 100 g of flour, 1.0 g of dry yeast, and 50 g of water. The selected addition levels of FBBF were determined by preliminary trials, and FBBF was incorporated into the dough at 0%, 0.5%, 1.0%, 1.5%, 2.0%, and 2.5% (*w*/*w*) of the total flour. Steamed bread prepared with *G. oregonense* black bean flour fermented for 40 d (FBBFSB) was made by a two-step fermentation method according to an existing procedure [26]. Similar to the co-fermentation strategy reported by Wang et al. (2025) for improving steamed bread quality, our two-step fermentation method also benefited from the synergistic effects of fungal and yeast activities [27].

### 2.11. FBBFSB Characteristics

Color: FBBFSB color was quantified by using Grating Spectrophotometer (YS3060, Shenzhen SUNSHI Technology Co., Ltd., Shenzhen, China), which was calibrated before use by using white and black standard tiles. The color parameters L*, a*, and b* were used as measures of lightness, redness/greenness, and yellowness/blueness, respectively. Three points per FBBFSB were measured.

Texture: The textural properties of FBBFSB were measured in the present study using the method proposed by Liang et al. (2022) [28].

Sensory evaluation: The sensory profile of FBBFSB was analyzed by a trained sensory panel (*n* = 10, aged 20–40 years old, 5 males and 5 females). Samples were coded with random three-digit numbers and presented in a randomized order. The sensory scores were assigned according to predefined evaluation criteria covering color, appearance, flavor, internal structure, taste, and overall acceptability. The analysis included five sensory attributes (color, appearance, flavor, taste, and acceptance). Please refer to Table S2 in the Annex for the specific sensory evaluation criteria. Mineral water (C’estbon; China Resources C’estbon Beverage Co. Ltd., China) was provided to cleanse the mouth before each sample evaluation.

### 2.12. Statistical Analysis

This study comprised two parts: the first involved the analysis of data from the fermentation process, including basic nutritional components (reducing sugars, protein, fat, ash, vitamin E and dietary fiber), β-glucosidase activity, bioactive compounds (total phenols, total flavonoids, total triterpenes) and Fourier-transform infrared spectroscopy; the second part involved the analysis of steamed bread prepared using whole mycelium products at varying addition levels, including measurements of color difference, texture, sensory evaluation and antioxidant activity.

All experiments were conducted in triplicate, and the results were expressed as mean ± standard deviation. Significant differences among samples were analyzed by one-way analysis of variance (ANOVA), followed by Duncan’s multiple range test, using IBM SPSS Statistics 25.0. Differences were considered significant at *p* < 0.05.

## 3. Results and Discussion

To better characterize the nutritional baseline of the substrate used in this study, the detailed composition of black bean is presented in Table S1. The results show that black bean is rich in crude protein, dietary fiber, and bioactive compounds, including total phenolics, flavonoids, γ-aminobutyric acid (GABA), and triterpenes, which provide a favorable nutritional and biochemical basis for solid-state fermentation.

In particular, the relatively high levels of phenolic and flavonoid compounds suggest strong inherent antioxidant potential, while the presence of GABA and triterpenes indicates the potential for further enhancement of functional properties during fungal fermentation. These compositional characteristics may contribute to the observed changes in antioxidant activity and bioactive compound accumulation during SSF.

### 3.1. Dynamic Changes in Ergosterol During SSF

**Figure 1:**
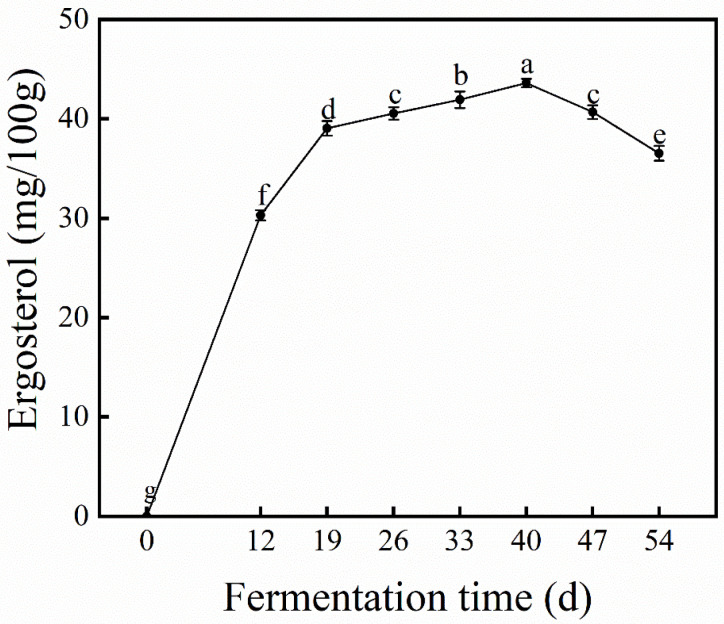
Ergosterol content of samples. Data are expressed as mean ± standard deviation (SD) (n = 3). Different letters (a–g) indicate significant differences among samples (*p* < 0.05).

As shown in Figure 1, ergosterol content in the first 19 d of SSF increased markedly, suggesting rapid biomass accumulation in the fermented black bean. Ergosterol is widely recognized as an indicator of fungal biomass [29,30]. After continuous growth from day 19 to day 40, the maximum ergosterol level (43.59 ± 0.43 mg/100 g) was observed on day 40. Further fermentation from 40 to 54 d resulted in a decrease in ergosterol content, suggesting that the fermentation of *G. oregonense* entered a later or decline phase. The reduction in ergosterol content may be associated with a decrease in microbial biomass and could involve multiple cellular processes, such as autophagy, apoptosis, or necrosis [30]. We therefore chose the first 40 days for dynamic monitoring of the nutritional components during the fermentation process.

The solid-state fermentation process of *Ganoderma oregonense* on black bean was divided into three stages according to the dynamic changes in ergosterol content and previous studies [29,31]. The period from 0 to 19 days is defined as the early fermentation stage, 19 to 33 days as the middle fermentation stage, and 33 to 40 days as the late fermentation stage. The early stage (0–19 d) could be further divided into two substages: 0–12 d, corresponding to the initial lag phase, and 12–19 d, corresponding to the rapid growth phase. The middle stage (19–33 d) was characterized by biomass accumulation, while the late stage (33–40 d) was associated with the transformation of amino acids and antioxidant components.

### 3.2. Basic Chemical Composition

**Figure 2:**
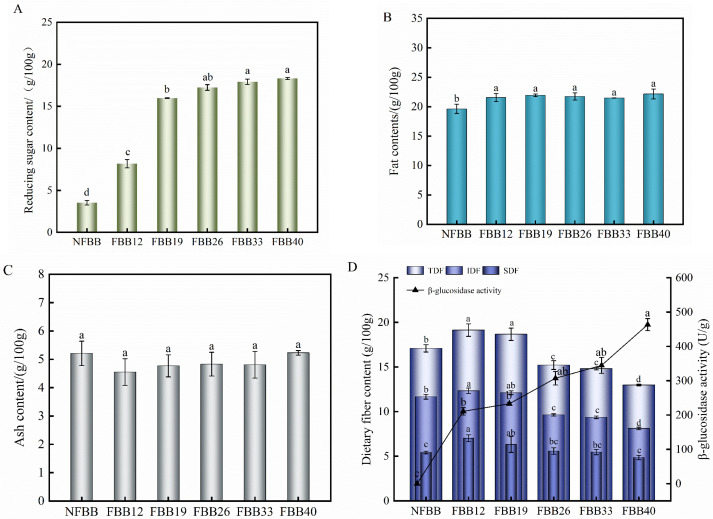
Changes in reducing sugar (**A**), fat (**B**), ash (**C**), dietary fiber, and β-glucosidase (**D**) content before and after fermentation. Different letters (a–f) indicate significant (*p* < 0.05) differences in the mean value. NFBB, non-fermented black bean; FBB12, FBB19, FBB26, FBB33, and FBB40, black bean fermented for 12, 19, 26, 33, and 40 d, respectively.

Changes in the reducing sugar content may reflect the degradation of reserve carbohydrates and structural polysaccharides during solid-state fermentation. Fermentation resulted in a significant 5.20 times increase in reducing sugar content (Figure 2A). Cen et al. (2024) [14] demonstrated that the fermentation of edible mushrooms can secrete large amounts of amylase and cellulase enzymes, which can hydrolyze macromolecules such as polysaccharides into oligosaccharides or monosaccharides, thereby increasing the release and bioaccessibility of small soluble sugars from the substrate. The crude fat content of the fermented samples was significantly higher than that of black bean flour, but the change with fermentation time was not statistically significant (*p* > 0.05) (Figure 2B). This increase may be due to the increased fatty acid content of black bean during fermentation, an observation similar to the results reported by Park et al. (2020) [32] for a study on natural fermentation of milled rice grains. Ash is the mineral element remaining after the carbon, hydrogen, oxygen, and nitrogen in the organic matter of the sample have been volatilized in the form of oxides. The observed increase in ash content (4.55–5.23 g/100 g) (Figure 2C) may be attributed to a relative concentration effect resulting from dry matter loss during fermentation (e.g., carbon dioxide release), which increases the apparent ash content on a dry weight basis. However, only slight numerical changes occurred due to the absence of other nutrients added before fermentation and the absence of leaching of minerals during the SSF fermentation, which did not cause significant differences following the fermentation time change [33].

### 3.3. Effect of Fermentation Time on the Content of Dietary Fiber (Df) and β-Glucosidase Activity

Figure 2D shows the changes in dietary fiber and β-glucosidase activity in black bean fermented with *G. oregonense* for 40 days. It was observed that black bean samples fermented with *G. oregonense* for 12 days exhibited the highest TDF (19.13 ± 0.70 g/100 g), SDF (7.02 ± 0.38 g/100 g) and IDF (12.11 ± 0.31 g/100 g). However, with increasing fermentation time (12 d to 40 d), TDF, SDF, and IDF content decreased gradually. Zhang et al. (2022) [34] provided a plausible explanation for this change: polysaccharides, such as β-glucan and arabinoxylan, were produced during fungal fermentation of substrates, leading to elevated levels of dietary fiber. In other words, before 12 d, *Ganoderma* mycelium growth remained limited, corresponding to the lag phase. At 12 d, *Ganoderma* mycelium began to extensively colonize the substrate, contributing to the accumulation of polysaccharides and lignin in FBB12. With the increase of fermentation time (12 d to 40 d), the β-glucosidase activity gradually rose and acted on the intramolecular β-D-glucose bonds to degrade macromolecules, resulting in a gradual decrease in DF content. DF, especially SDF, plays a key role in promoting intestinal flora balance and maintaining human health [35]. In this study, the SDF/IDF ratio in black bean fermented for 40 d significantly increased from 46.50% to 59.90% compared with that before fermentation. This suggests that *G. oregonense* can effectively degrade IDF to SDF and improve the bioaccessibility of the substrate.

### 3.4. Protein and Amino Acid Analysis

**Figure 3:**
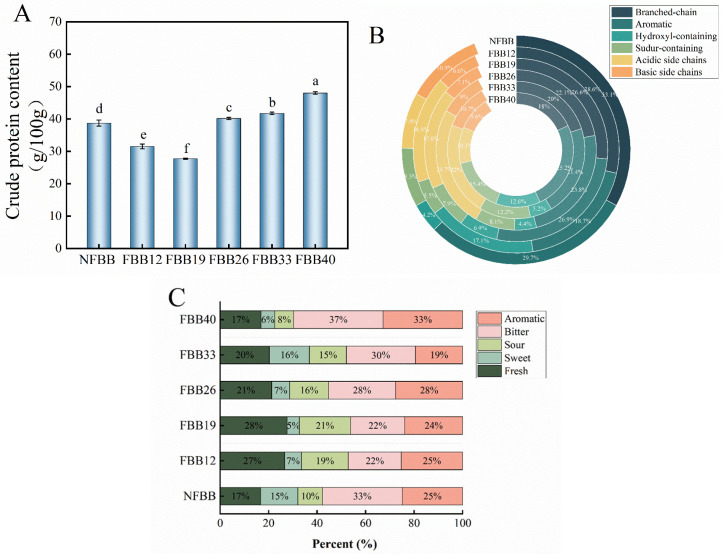
(**A**) Protein content of each sample. (**B**) Percentage contribution of amino acids during fermentation of *G. oregonense*, according to R groups: branched-chain (Val, Iso and Leu), aromatic (Phe and Tyr), hydroxyl-containing (Thr and Ser), sulfur-containing (Cys and Met), acidic side chains (Glu and Asp), and basic side chains (Lys and His). (**C**) Percentage contribution of amino acids during fermentation of *G. oregonense*, according to tasty: aromatic, bitter, sour, sweet, fresh.

Protein, the signature nutrient of black bean, was studied for its content changes during fermentation to understand the effect of different SSF times. Protein content showed an initial decrease followed by an increase during fermentation (Figure 3A). These changes were associated with the growth and metabolic activity of the fungus during SSF. According to Wang et al. (2023) [36], fungi may utilize protein as a nitrogen source to support growth during the lag phase (0–12 d). In general, the total nitrogen content of the system is relatively conserved during fermentation, although redistribution among different nitrogen fractions may occur. During the lag phase (0–12 d), macromolecular plant proteins are hydrolyzed into free amino acids by fungal extracellular enzymes [37]; minor losses may occur during Kjeldahl determination. At the same time, amino acids may be further metabolized through deamination reactions, and trace amounts of ammonia may be released if not fully assimilated by the system [38].

Furthermore, dissociation of bound nitrogen components in black beans (e.g., associated with phytic acid and tannins) may increase the proportion of water-soluble nitrogenous compounds available in the system, which may be partially incorporated into fungal biomass during early growth. After approximately 26 d of fermentation, the mycelium of *Ganoderma lucidum* exhibited rapid proliferation, indicating enhanced metabolic activity. Large amounts of cellulase were secreted to degrade dietary fiber and soluble sugars (non-nitrogenous carbohydrates), which were subsequently metabolized into CO_2_ and H_2_O and released into the environment. This process may contribute to dry matter loss and a relative increase in measured nitrogen content, leading to an apparent increase in protein content [39].

Several studies have shown that legumes, as a high-quality protein source, can be enriched through microbial SSF [7]. Fermentation with *Pleurotus ostreatus* increased the protein content of black bean by 6% [40]. In the present study, protein content in black bean increased by 24.07% after 40 days of fermentation compared with the unfermented sample. This increase may be attributed to both microbial biomass accumulation and compositional changes in the substrate during SSF.

The dynamics of the samples’ amino acid content before and after fermentation are shown in Table 1. The content of individual amino acids in black bean decreased significantly (*p* < 0.05) at the early stage of fermentation (0–12 d). At this stage, the rate of mycelial protein synthesis is significantly higher than the rate of substrate protein hydrolysis, resulting in a rapid decrease in the content of free amino acids in the sample. At 26 d of fermentation, the content of individual amino acids in fermented sample was significantly lower than the control sample (Table 1). This result was consistent with previous findings reported by Yang and Zhang (2009) [41] on soymilk fermentation using *Ganoderma lucidum*. At this fermentation stage, *G. oregonense* played the role of a consumer in consuming the amino acids in the substrate without supplying the sample with any additional free amino acids. However, it should be emphasized that the largest variety of amino acids was detected in the 33 d fermentation samples, and there was increased concentration of all amino acids between 26 d and 36 d fermentation, especially glutamic acid by a factor of 24.09. At this stage, the rate of mycelial protein synthesis may decrease, falling below the rate of plant protein hydrolysis, leading to the accumulation of free amino acids in the sample [42]. Some amino acids, such as Thr and Glu, decreased, while Val and Ser became undetectable after 33 days of fermentation. This phenomenon may be associated with enhanced amino acid catabolism during the late fermentation stage, in which branched-chain aminotransferase (BCAT) and related transaminases catalyze the conversion of amino acids into corresponding α-keto acids [43], which can be further metabolized via the tricarboxylic acid (TCA) cycle or utilized for the biosynthesis of volatile and nitrogen-containing compounds [44]. Meanwhile, extracellular proteases continuously hydrolyze proteins into peptides and free amino acids, resulting in a dynamic turnover of the nitrogen pool during solid-state fermentation [42]. This suggests that a proper fermentation time may harvest more kinds of amino acids. Consistent with previous studies [45,46], the total essential and free amino acids continued to increase and were elevated by 68.11% and 42.92%, respectively, during 40 d of fermentation. Concurrently, the EAA/TAA and EAA/NEAA ratios increased significantly, from 2.07 to 2.61, and 0.67 to 0.72, respectively, far exceeding the ideal protein model recommended by FAO/WHO (EAA/TAA > 0.4 and EAA/NEAA ratio > 0.6). However, since most of the samples were deficient in some essential amino acid species after fermentation, FBB33 is more in line with the ideal protein model proposed by WHO and FAO. Glu participates in the tricarboxylic acid cycle to synthesize GABA catalyzed by glutamate decarboxylase. Therefore, the change in GABA content in the later stages of fermentation (33–40 d) may be related to the reduction of glutamate content during fermentation.

Fungal fermentation produces the enzyme transaminase, which allows conversion between amino acids. The percentage of amino acids divided by R groups was analyzed to clarify the changes in amino acid side chain groups of black beans during fermentation and their potential health properties (Figure 3B). Branched-chain amino acids (BCAAs) are ideal compounds for use in foods and have many established health benefits including enhancing protein synthesis, controlling glucose metabolism, boosting immunity and slowing down the aging process [47]. Sulfur-containing amino acids synthesize antioxidants in cells [48]. The ratio of acidic to basic side chains may affect the pH of the matrix’s external environment, which in turn affects the release of actives [49]. As shown in Figure 3B, *G. oregonense* fermentation significantly altered the amino acid composition of black bean. In the early fermentation stage (12–19 d), the largest percentage of the FBBF samples were acidic side chains, followed by AAs and BCAAs. At 26–33 d of fermentation, the proportion of hydroxyl-containing amino acids increased while the proportion of AAs decreased. This may be the result of hydroxylation of aromatic amino acids [50]. At 40 d of fermentation, the content of BCAAs increased by 75% compared with that of the control group, and the proportion was the highest; the content of sulfur-containing and acidic side chains decreased significantly; and the content of aromatic amino acids, represented by Phe and Tyr, increased. These results indicated that *G. oregonense* fermentation significantly reshaped the amino acid profile of black bean and may contribute to improved nutritional quality.

**Figure 4:**
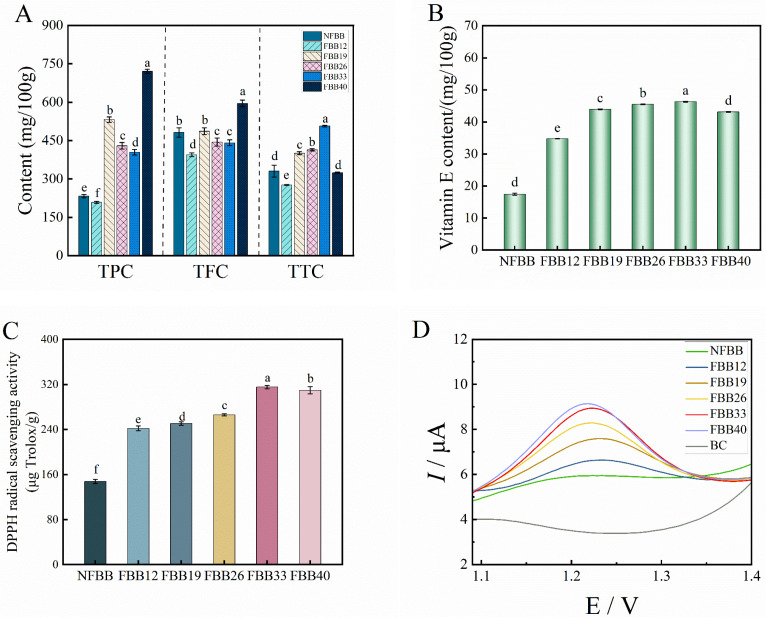
(**A**) Changes in total phenol (TPC), flavonoid (TFC) and total triterpene (TTC) content before and after fermentation. (**B**) Vitamin E content and (**C**) DPPH-radical antioxidant power of the NFBB and FBB. (**D**) Cyclic voltammogram of samples before and after fermentation. BC, blank control.

Flavor-presenting amino acids play a role in food flavor by enhancing taste and aroma. Different flavor-presenting elements help to create a more pleasing sensory experience in a wide range of foods, especially in fermented foods, where amino acids contribute significantly to sweet, fresh, or bitter flavors [51]. Figure 3C shows the compositional patterns of flavor-related amino acids in each sample. The results showed that all seven samples showed richer bitter and aromatic amino acids. The bitter amino acid percentage was significantly lower than that of the unfermented samples from 12–33 d of fermentation, while the bitter amino acid percentage of FBB40 at 40 d of fermentation was 4% higher than that of NFBB. In a previous study, Kaczmarska T. et al. (2018) [52] reported that bitter amino acids significantly increased after both natural fermentation of lupins and fermentation of soybean meal using *Lactobacillus delbrueckii* subsp. *bulgaricus* and *Streptococcus thermophilus*. This finding is consistent with the results of the present study [52]. Both fresh and sweet amino acids produce a pleasant flavor, while sour amino acids enrich the taste and balance the bitterness. As shown in Figure 4C, the overall percentage of the three amino acids in the fermented samples was higher than that of the NFBB, except for the FBB40 sample, after the fermentation treatment, which suggests that fermentation may have resulted in a more favorable organoleptic experience of black bean.

### 3.5. Changes in the Contents of Total Phenols, Flavonoids, and Triterpenes

The results of the changes in the total phenol content (TPC), total flavonoid content (TFC), and total triterpene content (TTC) of the samples before and after fermentation are shown in Figure 4A. Interestingly, during the SSF of black bean using *G. oregonense*, the contents of TPC and TFC increased by 2.28 and 1.23 times, respectively, from 12 d to 19 d, after which they decreased during the continuation of fermentation (19 d to 33 d). The increase in total phenolic and flavonoid contents during solid-state fermentation of black bean is consistent with the observations of Bo et al. (2026) [53] on black rice, where combined extrusion and fermentation also enhanced phenolic bioaccessibility. Some changes in external conditions (e.g., light, pH, temperature, etc.) may affect the solubility and bioaccessibility of phenolic compounds. As shown in Figure 3B, the ratio of basic and acidic side chains changed significantly during 12–33 d of fermentation, which may cause a change in the internal pH of the substrate, thus releasing some bound phenols that cannot be readily extracted from the substrate and improving the extractability of the phenols. TPC and TFC were detected at 40 d of fermentation with maximum concentration values of 720.65 ± 8.16 mg/100 g and 595.45 ± 13.16 mg/100 g, throughout the fermentation period. On the contrary, TTC increased significantly from 12–33 d and decreased after reaching a maximum of 506.45 ± 2.91 mg/100 g at 33 days of fermentation; it decreased or even fell slightly below that of the unfermented samples. A decrease in triterpene content was observed in the fermented Zanthoxylum pericardium residue of *G. lucidum* [33]. Terpenoids are formed by cyclization or modification of pentenyl diphosphate (IPP) and its isomer dimethylallyl diphosphate (DMAPP). IPP and DMAPP, which act as precursors produced from acetyl-CoA via the mevalonate pathway, produce steroids via the isoprene pathway. The decrease in triterpene content exhibited in this study may be related to more active enzymes produced by *G. oregonense* that would reduce its synthesis or convert it into other active components. The vitamin E (VE) content reached a maximum at 33 d (46.32 ± 0.13 mg/100 g) (Figure 4B). VE is a fat-soluble vitamin with strong antioxidant properties and excels in blocking lipid peroxidation and scavenging peroxyl radicals. The decrease in VE content caused by further prolongation of fermentation may be caused by oxidation of the active group of vitamin E or depletion by the *G. oregonense* metabolism.

### 3.6. Antioxidation Assay

The presence of antioxidants in beans can help to inhibit oxidative reactions by scavenging free radicals and minimizing the formation of reactive oxygen species, thereby preventing various health problems associated with oxidative stress [54]. The DPPH free-radical scavenging capacity of the samples at each fermentation stage was significantly influenced by *G. oregonense*. As shown in Figure 4C, the DPPH-radical scavenging activity in black bean increased rapidly after *G. oregonense* fermentation, then decreased, and increased by 2.13 times at 33 d of fermentation compared to before fermentation. A similar increase was observed in Tartary buckwheat fermented with *G. lucidum* [14]. Due to the different pretreatment of the samples, Li et al. (2023) [13] observed that DPPH-radical scavenging increased 9.43 times in the fermentation of black bean with *G. oregonense* for 35 d. It should be noted that the TPC of FBB40 was higher than the other samples, but the free-radical scavenging activity of DPPH was lower than that of FBB33. Synergistic or antagonistic effects exist between antioxidants including tocopherols (vitamin E), ascorbic acid (vitamin C), carotenoids, and polyphenolic compounds (e.g., flavonoids and phenolic acids) in foods [55]. The change of phenolic compounds and DPPH-radical scavenging rate in the samples does not fully reflect their biological significance. So, we further evaluated the antioxidant capacity of the samples using electrochemical methods. In recent years electrochemical sensors have become a promising alternative for assessing the antioxidant capacity of complex samples due to their cost-effectiveness [33,56]. Figure 4D displays cyclic voltammograms of black bean at different stages of fermentation. From the figure, it can be seen that all the fermented sample solutions showed obvious oxidation peaks near the potential 1.2 V. The longer the fermentation time, the higher the oxidation peak current of the extracts, indicating that the concentration of antioxidants in the samples increased, and their antioxidant capacity was enhanced. This is consistent with our previous findings on *G. oregonense*-fermented germinated black bean, further validating that this new antioxidant component is due to the fermentation of *G. oregonense* [23]. Overall, fermentation had a positive effect on the antioxidant capacity of black bean; meanwhile, the differences between the pre-treatments applied to the black bean highlight the importance of the process variables used during fermentation.

### 3.7. FTIR Analysis

**Figure 5:**
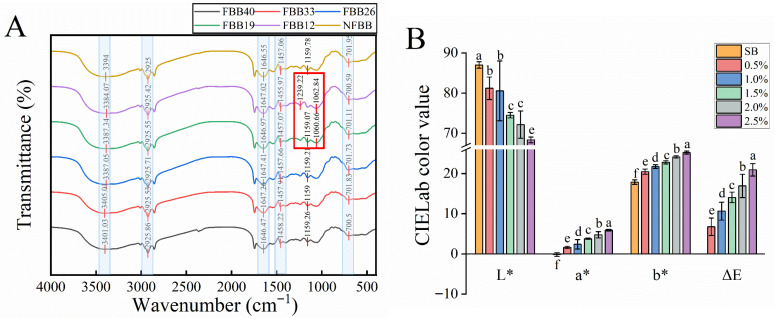
(**A**) Fourier infrared spectra of samples before and after fermentation. (**B**) Color results of different steamed bread. Note: SB denotes steamed bread without added FBBF, and i% denotes steamed bread with i% FBB40 flour.

FTIR analyses showed that the functional group characteristics of the samples were dominated by characteristic bands of cellulose, hemicellulose, and lignin (Figure 5A) [57]. All samples showed peaks near 3400, 2925, 1650, 1460, and 700 cm^−1^, with slight shifts in the peaks of these characteristic peaks. The absorption peaks of the samples around 3400 cm^−1^ were mainly derived O-H and N-H stretching mainly from cellulose and hemicellulose, and the area and intensity of the absorption peaks increased with the increase of the fermentation time, which may imply that a large number of hydroxyl radicals were released by the fermentation of *G. oregonense*. The 2925 cm^−1^ peak observed is attributed to the C–H bond stretching of the polysaccharide. The absorption peak near 1650 cm^−1^ is related to the C–O tensile vibration in the lignin benzene ring. The band located in the range of 1640–1650 cm^−1^ can be used to characterize the irregular curling of proteins. The absence of large differences in the absorption peaks occurring in this range during fermentation may indicate that the fermentation of *G. oregonense* did not cause a large degree of disruption of the protein molecules as a result of the fermentation, and therefore it is hypothesized that the large number of free amino acids produced in the later stages of the fermentation may have been caused by the high concentration of biomass [58]. The appearance of the 1450 cm^−1^ peak was due to the asymmetric bending vibration of the methyl group. The peak at 700 cm^−1^ arises due to the C–H bending of four or more methylene groups. Particularly, FBB12 and FBB19 have increased absorption peaks near 1100 cm^−1^. According to Chylińska et al. (2016) [59], the samples enriched in glucuronic acids (e.g., galacturonic acid) show strong absorption peaks in the range 1100–1018 cm^−1^. This may indicate an increase in glucuronic acid content during the 12–19 d period of *G. oregonense* fermentation of black bean, which corroborates the results in Section 3.2 regarding the determination of dietary fiber content.

### 3.8. Effect of FBBF on Quality of SB

#### 3.8.1. Color and Texture Properties of SB

Color gives the most intuitive feeling of food and plays a decisive role in the consumption and popularity of the product [60]. Figure 5B shows the color characteristics of SB made by replacing different percentages of wheat flour with FBB40 flour. As expected, the addition of dark flour significantly affected the color of SB. As the amount of added FBBF increased, the L* value of FBBSB decreased, while the a* value and b* value increased, but the b* value was always much higher than the a* value. Therefore, FBBSB is yellowish. As yeast metabolism reduces the oxidation of colored compounds, the more FBBF was doped, the higher the ΔE value of FBBFSB relative to SB. This is consistent with the findings reported by Chen R. et al. (2022) on steamed bread prepared with different levels of red beetroot powder [61]. Texture is also an important physical indicator for evaluating the palatability of food products [62]. Therefore, we analyzed the textural characteristics of FBBF-added steamed bread (Table 2). The hardness of the FBBFSB reached the maximum value at 0.5% of the added amount and gradually decreased with the increase of the amount of FBBF added. Larger fiber particle sizes are associated with harder texture [63]. The decrease in hardness values may be related to the degradation of dietary fiber during yeast fermentation. Bai et al. (2024) reported similar observations of lower hardness values of steamed bread prepared with added red kidney bean polysaccharides [64]. Replacing 1.0% wheat flour with FBB40 flour showed better performance in terms of cohesiveness and springiness. However, there were significant differences (*p* < 0.05) between 1.0% FBBF and 1.5% FBBF in aspects of adhesiveness, cohesiveness, springiness, gumminess, and chewiness. This may be caused by a change in the secondary structure of the protein in the dough [65]. FBBSB has less gumminess and chewiness than wheat SB, which is more suitable for infants, young children, and the elderly [64].

#### 3.8.2. Sensory Evaluation of SB

**Figure 6:**
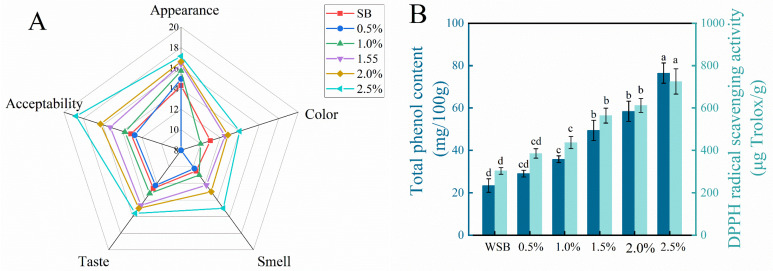
(**A**) Sensory evaluation. (**B**) Total phenolic content and DPPH-radical scavenging activity results of different steamed bread. Note: SB denotes steamed bread without added FBBF, and i% denotes steamed bread with i% FBB40 flour.

The results of sensory evaluation on SB containing FBBF are given in Figure 6A. SB with added FBBF outperformed SB in all aspects. Among all FBBFSB, SB with 2.5% FBBF received the highest ratings for appearance, color, smell, taste, and acceptability. As noted by Awulachew (2025) for bread containing fava bean flour, the sensory acceptability of steamed bread in this study remained favorable at low to moderate substitution levels (≤2.5%) [66]. A 2.5% FBBFSB is blown up at the top, with a flower-like outer shape; is light yellow; and has the characteristic aroma of FBBF and a soft texture. It will be necessary to analyze the specific aroma components and the taste characteristics using some scientific instruments in the future. The nutritional properties of FBBFSB were not studied in detail, as the nutrient content of the raw material can be retained in large quantities through simple processing. However, there is a need to optimize the preparation process to increase nutrient intake and maintain consumer sensory acceptability.

#### 3.8.3. Total Phenolic Content and DPPH-Radical Scavenging Capacity of SB

As shown in Figure 6A, the addition of FBB40 increased the total phenol content and DPPH-radical scavenging rate of FBBSB with an increasing trend with the increasing amounts of additions. Similarly, P. Li et al. (2024) [67] found that buns prepared with the addition of fish skin collagen peptide showed a dose-dependent increase in both ABTS and DPPH-free-radical scavenging rates in the 2.0% addition range. The total phenol and DPPH-radical scavenging rates of SB prepared with 2.5% FBB40 were 3.26 and 2.39 times higher than those of SB, respectively. Similar to the findings of Lin et al. (2025) [68] for rice sourdough, the addition of fermented black bean flour also enhanced the total phenolic content and radical scavenging capacity of steamed bread. Therefore, the addition of FBB40 can effectively improve the antioxidant property of composite steamed bread.

## 4. Conclusions

In this study, solid-state fermentation of black bean using *G. oregonense* was carried out to investigate the changes in various nutrients and antioxidant activities within black bean at different fermentation times. The fermentation process lasted for 40 days, resulting in a decrease in the dietary fiber content of FBB, which was converted into a more bioavailable form. Meanwhile, the content of reducing sugars, total phenols, total flavonoids and VE increased. The antioxidant capacity of black beans increased with fermentation time. *G. oregonense* fermentation induced significant changes in small-molecule amino acids and proteins, making it a sustainable source of high-quality protein. In the early stage of fermentation (0–19 d), corresponding to the lag and rapid growth phases, macromolecular plant proteins were progressively hydrolyzed into free amino acids by fungal extracellular enzymes. However, these amino acids were rapidly utilized by the growing mycelium as nitrogen sources for biomass synthesis, resulting in a decrease in free amino acid content. During the middle stage (19–31 d), fungal growth and metabolic activity reached a maximum level, accompanied by enhanced degradation of carbohydrates and other non-nitrogenous components. In the late stage (31–40 d), the rate of plant protein hydrolysis exceeded fungal utilization, leading to the accumulation of free amino acids. Meanwhile, autolysis of fungal biomass further contributed to the release of intracellular nitrogen-containing compounds, promoting the transformation of amino acids and antioxidant components. FBB40 flour was used as a potential functional food ingredient to partially replace wheat flour to prepare a novel steamed bread with good organoleptic quality, demonstrating the feasibility of using flour made from black bean treated with *G. oregonense* fermentation to produce products of good quality. However, detailed studies on the other metabolites during the fermentation process are necessary to clarify the precise mechanisms that lead to the enhanced nutritional value of black bean and other potential health benefits. In addition, more research is needed to identify the optimum fermentation conditions to maximize the release and utilization of its nutritional value. In conclusion, functional food development using *G. oregonense*-fermented black bean flour as a high-value raw material seems profitable.

## Figures and Tables

**Table 1:** Amino acid levels in fermented black bean as a function of fermentation time.

FAA		Free AA Content (g/100 g Protein)					
		NFBB	FBB12	FBB19	FBB26	FBB33	FBB40
Essential amino acids (EAAs)	Thr	8.43 ± 0.28 ^b^	ND	ND	ND	8.82 ± 0.12 ^a^	5.27 ± 0.04 ^c^
	Val	2.03 ± 0.12 ^b^	0.14 ± 0.00 ^c^	ND	ND	6.06 ± 0.08 ^a^	ND
	Met	12.81 ± 0.28 ^a^	0.16 ± 0.01 ^e^	0.41 ± 0.01 ^cd^	0.56 ± 0.03 ^d^	3.57 ± 0.20 ^c^	5.74 ± 0.15 ^b^
	Ile	4.62 ± 0.37 ^c^	0.19 ± 0.01 ^f^	0.75 ± 0.01 ^e^	1.38 ± 0.08 ^d^	9.80 ± 0.15 ^b^	11.80 ± 0.09 ^a^
	Leu	8.30 ± 0.27 ^c^	0.36 ± 0.01 ^f^	1.46 ± 0.12 ^e^	2.26 ± 0.13 ^d^	11.21 ± 0.13 ^b^	17.85 ± 0.82 ^a^
	Phe	6.80 ± 0.10 ^c^	0.39 ± 0.02 ^f^	1.28 ± 0.04 ^e^	1.93 ± 0.08 ^d^	11.44 ± 0.27 ^b^	16.33 ± 0.16 ^a^
	His	2.42 ± 0.03 ^a^	0.09 ± 0.00 ^d^	0.14 ± 0.00 ^d^	0.18 ± 0.00 ^d^	1.36 ± 0.17 ^c^	2.06 ± 0.08 ^b^
	Lys	5.55 ± 0.51 ^c^	0.28 ± 0.01 ^e^	0.76 ± 0.01 ^d^	0.79 ± 0.01 ^d^	6.99 ± 0.12 ^b^	11.65 ± 0.09 ^a^
	Total	51.00 ± 0.71 ^c^	1.65 ± 0.04 ^f^	4.83 ± 0.21 ^e^	7.12 ± 0.15 ^d^	59.28 ± 0.24 ^b^	70.74 ± 0.29 ^a^
Non-essential amino acid (NEAAs)	Asp	3.87 ± 0.04 ^a^	0.34 ± 0.01 ^e^	1.32 ± 0.06 ^d^	1.71 ± 0.01 ^c^	3.63 ± 0.02 ^b^	3.90 ± 0.06 ^a^
	Ser	2.03 ± 0.03 ^b^	0.18 ± 0.01 ^e^	0.44 ± 0.01 ^d^	0.94 ± 0.01 ^c^	12.75 ± 0.04 ^a^	ND
	Glu	4.64 ± 0.04 ^c^	0.42 ± 0.01 ^f^	1.15 ± 0.01 ^d^	0.73 ± 0.01 ^e^	10.59 ± 0.07 ^a^	5.09 ± 0.08 ^b^
	Cys	ND	0.26 ± 0.02 ^c^	0.40 ± 0.02 ^c^	0.52 ± 0.03 ^c^	3.33 ± 0.80 ^b^	3.75 ± 0.12 ^a^
	Tyr	14.08 ± 0.71 ^b^	0.35 ± 0.02 ^e^	1.10 ± 0.03 ^d^	1.75 ± 0.12 ^d^	8.18 ± 0.10 ^c^	15.06 ± 0.70 ^a^
	Total	24.63 ± 0.64 ^c^	1.56 ± 0.02 ^f^	4.43 ± 0.03 ^e^	5.67 ± 0.09 ^d^	38.48 ± 0.73 ^a^	27.81 ± 0.74 ^b^
Total		75.64 ± 0.56 ^c^	3.22 ± 0.02 ^f^	9.27 ± 0.19 ^e^	12.79 ± 0.21 ^d^	97.77 ± 0.91 ^b^	98.56 ± 0.97 ^a^
γ-aminobutyric acid (GABA) (mg/100 g)		27.03 ± 0.53 ^d^	35.54 ± 0.47 ^c^	45.41 ± 0.07 ^b^	47.26 ± 0.83 ^b^	52.23 ± 0.71 ^a^	36.56 ± 0.45 ^c^

Values in the same line with different letters differ significantly (*p* < 0.05). NFBB, non-fermented black bean; FBB12, FBB19, FBB26, FBB33, and FBB40, black bean fermented for 12, 19, 26, 33, and 40 d, respectively; ND, not detected.

**Table 2:** Textural properties of steamed bread with different additive levels.

Sample	Hardness (N)	Adhesiveness	Cohesiveness	Springiness (mm)	Gumminess (N)	Chewiness (mJ)
SB	5.17 ± 0.39 ^b^	0.99 ± 0.15 ^c^	0.71 ± 0.03 ^a^	12.65 ± 0.21 ^a^	3.78 ± 0.30 ^a^	46.68 ± 3.85 ^a^
0.5%	6.18 ± 0.70 ^a^	0.36 ± 0.09 ^d^	0.46 ± 0.05 ^c^	11.05 ± 0.73 ^bc^	3.07 ± 0.54 ^b^	29.97 ± 4.06 ^c^
1.0%	4.61 ± 0.34 ^c^	1.11 ± 0.31 ^bc^	0.69 ± 0.07 ^a^	12.79 ± 0.97 ^a^	3.21 ± 0.21 ^b^	41.21 ± 3.91 ^b^
1.5%	4.27 ± 0.44 ^c^	3.31 ± 0.47 ^a^	0.46 ± 0.11 ^c^	10.63 ± 0.55 ^c^	2.04 ± 0.59 ^c^	24.10 ± 6.00 ^d^
2.0%	3.57 ± 0.23 ^d^	1.29 ± 0.32 ^bc^	0.56 ± 0.09 ^b^	11.72 ± 0.90 ^b^	2.27 ± 0.30 ^c^	25.86 ± 5.93 ^cd^
2.5%	3.48 ± 0.33 ^d^	1.35 ± 0.06 ^b^	0.62 ± 0.08 ^ab^	11.56 ± 1.07 ^b^	2.34 ± 0.41 ^c^	28.50 ± 5.46 ^cd^

The fermented and unfermented samples were conducted in triplicate for each indicator.

## Data Availability

All data supporting this study are included in this manuscript. Further details are available from the corresponding authors upon request.

## Supplementary Materials

The following supporting information can be downloaded at: https://www.mdpi.com/article/10.3390/foods15091588/s1, Table S1. Nutritional composition of black bean; Table S2. Sensory evaluation criteria for steamed bread.
