# Supplementary material for: Enhancing the Quality of Black Bean by Ganoderma oregonense Solid-State Fermentation and Its Application in Steamed Bread
Source: Foods. 2026 May 4;15(9):1588. doi: 10.3390/foods15091588 (PMC13163655; doi:10.3390/foods15091588)
Supplement: Supplementary file 1 [file foods-15-01588-s001.zip › foods-4250793-supplementary.pdf]

Table S1. Nutritional composition of black bean

Parameter	Value	Unit
Reducing sugar content	3.5227±0.157	g/100 g
Dietary fiber content	16.8±0.23	%
Vitamin E content	17.4286±0.561	mg/100 g
Ash content	5.21±0.421	%
Fat content	19.64±0.359	%
Crude protein content	387.1880±5.13	mg/g
Total triterpene content	330.2535±3.617	mg/100 g
γ-Aminobutyric acid (GABA) content	27.0375±0.411	mg/100 g
Total antioxidant capacity	15.6300±0.322	(μmol/g)
Total phenolic content (TPC)	232.873±3.441	mg/100 g
Total flavonoid content (TFC)	481.5112±6.1227	mg/100 g
DPPH radical scavenging activity	147.9782±2.8511	μg Trolox/g

* Values are expressed as mean ± standard deviation (n = 3).

* Crude protein content was calculated from total nitrogen using a conversion factor of 6.25.

Table S2. Sensory evaluation criteria for steamed bread.

Evaluation item	Scoring criteria
Color (15 points)	Uniform color and normal appearance (11–15 points); relatively uniform color with slightly dull appearance (6–10 points); dark, uneven color without gloss (0–5 points).
Appearance (20 points)	Good external appearance; plump surface; flat base; no shrinkage (15–20 points); Fair external appearance; plump surface; uneven base; no shrinkage (8–14 points); Poor external appearance; not plump; uneven base; bubbles or shrinkage (0–7 points).
Smell (25 points)	Distinct aroma characteristic of fermented black bean flour and fermented wheat products, with high intensity (15–20 points); similar aroma but with lower intensity (8–14 points); weak aroma or presence of off-flavors (0–7 points).
Taste (20 points)	Moderate hardness, pure and slightly sweet taste, non-sticky (11–20 points); relatively soft texture, either overly sweet or lacking sweetness, slightly sticky (6–10 points); overly sweet or cloying, difficult to swallow, and sticky (0–5 points).
Overall acceptability (20 points)	Soft and elastic, with good springiness upon compression and chewiness (12–20 points); relatively soft with moderate elasticity (6–11 points); dense with low elasticity (0–5 points).
